# State Quitlines and Cessation Patterns Among Adults With Selected Chronic Diseases in 15 States, 2005–2008

**DOI:** 10.5888/pcd9.120105

**Published:** 2012-11-08

**Authors:** Terry Bush, Susan M. Zbikowski, Lisa Mahoney, Mona Deprey, Paul Mowery, Barbara Cerutti

**Affiliations:** Author Affiliations: Susan M. Zbikowski, Lisa Mahoney, Mona Deprey, Alere Wellbeing (formerly known as Free & Clear), Seattle, Washington; Paul Mowery, Centers for Disease Control and Prevention, Atlanta, Georgia; Barbara Cerutti, Imperial College London, London, England.

## Abstract

**Introduction:**

The death rate of people who have a chronic disease is lower among former smokers than current smokers. State tobacco cessation quitlines are available for free in every state. The objective of our study was to compare demographic characteristics, use of quitline services, and quit rates among a sample of quitline callers.

**Methods**

We used data from 15 states on tobacco users aged 18 or older who enrolled with a quitline between October 1, 2005, and May 31, 2008; 9 states also provided data from 7-month follow-up surveys. We used descriptive statistics and logistic regression to compare callers by disease status.

**Results:**

Among 195,057 callers, 32.3% reported having 1 or more of the following chronic diseases: 17.7%, asthma; 5.9%, coronary artery disease; 11.1%, chronic obstructive pulmonary disease; and 9.3%, diabetes; 9.0% had 2 or more chronic diseases. Callers who had a chronic disease were older and better educated; more likely to be female, have Medicaid or other health insurance, and have used tobacco for 20 years or more; and less likely to quit smoking (22.3%) at 7 months than callers who had none of these chronic diseases (29.7%).

**Conclusion:**

About one-third of tobacco users who call state quitlines have a chronic disease, and those who have a chronic disease are less likely to quit using tobacco. Continued efforts are needed to ensure cessation treatments are reaching tobacco users who have a chronic disease and to develop and test ways to increase quit rates among them.

## Introduction

Each year cigarette smoking causes 443,000 deaths and $193 billion in health care expenditures and productivity losses in the United States (1,2). These costs are higher among people who have a chronic disease such as diabetes, cancer, or cardiovascular disease. In 2006, the age-adjusted prevalence of smoking was 36.9% among people who had a smoking-related chronic disease and 19.3% among those who had other chronic diseases or had no chronic disease (3,4). Smoking is an independent and modifiable risk factor for type 2 diabetes (5), heart disease (6), and chronic obstructive pulmonary disease (COPD) (7). An estimated 90% of deaths from COPD are directly attributable to smoking; quitting is the only proven way to stop progression of the disease (7). Smoking has also been shown to increase the onset and severity of asthma and heart disease (6,8). One cost-effective population-based strategy to increase smoking cessation is proactive telephone counseling (9–12). In the United States, a toll-free national number (1-800 QUITNOW) connects callers to their state quitline for services such as mailed materials, proactive counseling, and mailed nicotine replacement therapy (NRT) (patch or gum). Quitlines provide a brief, minimally invasive, convenient form of cessation treatment, reach diverse segments of the population, and may be especially useful for chronically ill people who have limited mobility (9). The option of receiving services over the telephone increases the use of cessation treatments (13).

Little is known about the use of quitlines and quit rates among tobacco users who have a chronic disease. A few studies have described the characteristics of people who use quitlines but did not describe the prevalence of chronic disease among their study populations (14). The objective of our study was to compare the characteristics, use of quitline services, and quit rates among a sample of quitline callers. This information will provide an understanding of how state quitlines are serving tobacco users who have a chronic disease and help to determine whether treatment should be modified for this population.

## Methods

Alere Wellbeing conducted this observational study. Alere is the largest provider of quitline services in the United States, serving more than 350,000 tobacco users annually. This study was approved by the Western Internal Review Board.

### Sample

Fifteen state quitlines operated by Alere at the time of the study (Alaska, Connecticut, Georgia, Hawaii, Indiana, Maryland, Missouri, North Carolina, Oklahoma, Oregon, South Carolina, Utah, Virginia, Washington, and Wisconsin) agreed to participate and allowed use of data routinely collected from quitline callers. We included data if the caller was a current tobacco user, was aged 18 or older, and had enrolled in a state quitline between October 1, 2005, and May 31, 2008. We selected this period so that we could include data from 7-month follow-up telephone surveys. We excluded data on callers who were pregnant or did not have a reliable telephone number. The final sample included 195,057 callers. The standard cessation benefits offered to all callers, regardless of readiness to quit, were a single “reactive” counseling call and mailed materials. For callers ready to quit within 30 days (and only these callers), the quitline offered a multicall program, consisting of the standard reactive counseling call plus 3 or 4 proactive counseling calls and NRT. Of the 195,057 callers, 128,997 (66.1%) were enrolled in the multicall program. All but 2 states (North Carolina and Virginia) provided free NRT (2 or 4 weeks) for selected populations (eg, uninsured callers). State services varied according to funding available for tobacco control programs (15).

Nine state quitlines also provided data from telephone surveys conducted 7 months after enrollment with randomly selected callers. These follow-up surveys were conducted by an external evaluation service between April 1, 2006, and December 31, 2008. Response rates ranged from 34% to 52%, and 4,730 callers completed the survey; 68.7% (n = 3,250) were enrolled in the multicall program. Callers provided verbal consent before administration of the survey.

### Measures

Quitlines routinely collect information from callers on demographics and tobacco use, quitline services used (whether counseling only or counseling and NRT and number of counseling calls completed), and quit outcomes. For this study, we used data on caller demographics (age, sex, race/ethnicity, education, and insurance status), current tobacco use, tobacco type used (cigarettes vs other), years of tobacco use (<20 years vs ≥20 years), amount used (eg, cigarettes per day), time to first tobacco use after waking (a brief measure of addiction [16]), whether they live with or work with smokers, and how they heard about the quitline. To assess whether a caller had a chronic disease, quitline telephone staff asked, “Have you been diagnosed with any of the following conditions: asthma, chronic obstructive pulmonary disease or emphysema, coronary artery disease (CAD), or diabetes?” The 7-month follow-up survey asked about use of NRT since enrolling with the quitline and last use of tobacco since enrolling (≥7 days and ≥30 days). These measures have been used in other quitline studies (17).

### Analyses

We used frequencies and means to describe demographic characteristics and χ^2^ tests or analysis of variance to compare demographics, the types of quitline services used, and abstinence by chronic disease status (any chronic disease vs none of the 4 diseases) and for each disease separately (eg, asthma only vs no asthma). We defined “any chronic disease” as having 1 disease or a combination of diseases. We also compared the demographic characteristics of the entire sample of callers with the callers who participated in the 7-month telephone survey.

Using complete case analyses of the 7-month survey data (n = 4,730), we conducted multiple logistic regression to determine whether chronic disease was associated with 30-day abstinence. We repeated these analyses for 7-day abstinence. To inform our selection of covariates to be included in regression models, we performed correlation analyses to determine colinearity of independent variables. As expected, age was correlated with years of tobacco use (*r* = 0.85); time to first tobacco use after waking was correlated with cigarettes per day (*r* = 0.41); and race was correlated with ethnicity (*r* = 0.37). Because age was more strongly associated with abstinence than years of tobacco use, we used age in the logistic regression models. We included all of the covariates in the initial regression model and then used backwards elimination to remove variables that were not significant (*P* ≥ .05). The final model included chronic disease status (variable of interest), age, sex, race, education, insurance status, number of cigarettes smoked per day, and state (entered to control for state variability in treatment offerings). The final model used chronic disease as a 2-level predictor (any of the 4 diseases vs no disease) and then as a 6-level variable (asthma only, CAD only, COPD only, diabetes only, ≥2 diseases, or no disease). We repeated this analysis with callers in the multicall program (n = 3,250) and added number of counseling calls completed and receipt of NRT to determine the association between chronic disease and abstinence after controlling for these factors. Analyses were conducted in SAS version 9.1.3 (SAS Institute, Cary, North Carolina).

## Results

Among 195,057 tobacco users who enrolled with 1 of the 15 state quitlines between October 1, 2005, and May 31, 2008, 32.3% reported having at least 1 of the 4 chronic diseases; 17.7% had asthma (11.2%, asthma only), 5.9%, CAD (2.4%, CAD only); 11.1% COPD (4.8%, COPD only); and 9.3%, diabetes (4.9% diabetes only). These proportions include some duplication; 17,609 callers (9.0%) had 2 or more chronic diseases; 132,033 (67.7%) had none of the 4 diseases, and 45,415 (23.3%) had only 1 chronic disease. Among the 9.0% who had 2 or more chronic diseases, 66.5% had asthma.

In bivariate analyses, callers who had any chronic disease differed significantly from callers who had none (Table 1) by age, sex, education, insurance status, how long they used tobacco, exposure to smokers at home or work, time to first tobacco use after waking, and how they heard about the quitline. Callers also differed significantly by type of chronic disease. For example, compared with callers in the other 3 single disease groups, callers who had asthma only were younger, callers who had COPD only were more likely to be non-Hispanic white, and callers who had COPD only or 2 or more chronic diseases were more likely to use tobacco within 5 minutes of waking. The callers who participated in the 7-month follow-up survey had similar characteristics.

**Table 1:** Characteristics of Quitline Users (N = 195,057) by Chronic Disease Status, 15 State Quitlines, October 1, 2005, through May 31, 2008

Characteristic	Any Chronic Disease^a^	No Chronic Disease^a^	Asthma Only	CAD Only	COPD Only	Diabetes Only	≥2 Chronic Diseases^b^
**Callers, n (%)**	63,024 (32.3)	132,033 (67.7)	21,928 (11.2)	4,598 (2.4)	9,407 (4.8)	9,482 (4.9)	17,609 (9.0)
**Age, mean (SD), y**	46.0 (14.0)	39.1 (13.1)	36.3 (12.3)	53.8 (11.6)	52.7 (11.5)	48.6 (12.4)	51.0 (11.6)
**Age group, y**							
18–24	8.8	15.9	21.0	1.4	1.4	3.6	2.4
25–44	33.6	48.4	51.7	18.0	19.7	31.3	23.6
45–64	48.8	32.3	25.6	63.2	64.1	55.4	62.3
≥65	8.8	3.4	1.7	17.4	14.8	9.7	11.7
**Sex**							
Female	67.1	57.1	71.0	49.3	64.5	60.1	72.0
**Race/ethnicity**							
White non-Hispanic	76.6	76.2	74.3	78.6	86.0	65.9	79.6
African American non-Hispanic	13.2	13.6	13.6	13.7	7.4	22.9	10.7
Native American or Alaska Native non-Hispanic	5.2	3.8	5.4	4.6	4.4	4.9	6.0
Asian non-Hispanic	0.7	1.2	0.9	0.4	0.2	1.2	0.5
Hispanic	4.2	5.2	5.9	2.8	2.0	5.1	3.3
**Education**							
≤High school	60.6	56.7	60.8	59.3	62.9	56.4	61.7
**Insurance status**							
Insured	42.3	43.0	34.2	55.0	46.6	49.2	43.0
Medicaid	28.3	15.8	27.0	20.3	25.8	24.2	35.6
Uninsured	29.4	41.6	38.8	24.8	27.6	26.7	21.4
**Exposure to smokers**							
Smokers at home	39.6	30.5	38.7	36.3	39.5	35.1	44.0
Smokers at work	9.8	18.2	13.0	10.0	8.4	12.1	5.6
Smokers at home and work	11.8	18.7	17.8	10.2	8.7	10.8	7.3
**Tobacco use**							
Used ≥20 y	74.6	54.3	48.6	92.1	92.5	80.8	89.6
First use within 5 min of waking	56.7	47.7	54.4	52.8	60.1	51.8	61.7
No. of cigarettes per day, mean (SD)	21.8 (13.6)	19.4 (11.8)	20.3 (12.2)	20.5 (13.1)	23.3 (14.1)	21.2 (13.3)	23.4 (14.8)
**How caller heard of quitline**							
Health care professional	17.7	10.9	14.7	20.3	17.5	17.5	21.1
Family or friend	18.8	22.0	21.8	15.9	18.1	17.2	16.9
Radio or television	26.4	32.4	28.4	25.8	26.5	28.9	22.7
Other	37.1	34.7	35.1	38.0	37.9	36.5	39.3
**Enrollment type**							
In multicall counseling program	66.3	66.0	66.9	63.6	67.2	64.9	66.8
**Services used by callers enrolled in the multicall counseling program (n = 128,997)**							
No. of calls completed, mean (SD)	2.6 (1.8)	2.4 (1.4)	2.4 (1.6)	2.7 (1.6)	2.8 (1.7)	2.7 (1.9)	2.9 (1.9)
Mailed nicotine replacement therapy	63.7	69.8	68.6	54.6	65.6	62.1	59.6

Abbreviations: CAD, coronary artery disease; COPD, chronic obstructive pulmonary disease.

^a^ In bivariate comparisons of “any chronic disease” group and the “no disease group,” all differences were significant at *P* < .001. The survey asked about the following 4 chronic diseases only: asthma, CAD, COPD, and diabetes. All values are percentages unless noted.

^b^ In 6-level comparisons (the “no disease” group, asthma only, CAD only, COPD only, diabetes only, and ≥2 chronic disease), all differences were significant at *P* < .001.

The mean number of calls completed among callers in the multicall program ranged from 2.4 calls to 2.9 calls and differed significantly between disease groups (any vs none, asthma only vs no asthma, etc.). Similarly, the percentage of callers in the multicall program who received NRT ranged from 54.6% to 69.8% and differed significantly between disease groups.

In unadjusted analyses of 7-day and 30-day quit rates, callers who had any chronic disease were less likely to quit tobacco than callers who had none of these chronic diseases (Figure). The 30-day quit rate was 22.3% among callers who had any chronic disease and 29.7% among callers who had none of these diseases. Quit rates also differed significantly between callers who had none of these chronic diseases and callers in each of the 4 disease groups (*P* < .001) and callers who had 2 or more chronic diseases (*P* < .001).

**Figure:**
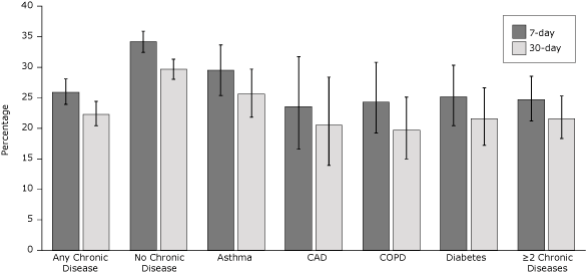
Unadjusted quit rates by chronic disease status among the 4,730 callers who completed the 7-month survey; 68.7% of survey completers were enrolled in the multicall program. Error bars represent 95% confidence intervals. Abbreviations: CAD, coronary artery disease; COPD, chronic obstructive pulmonary disease. **Table:** 

Abstinence Group	Unadjusted Quit Rates, % (95% Confidence Interval)						

	Any Chronic Disease	No Disease	Asthma	Coronary Artery Disease	Chronic Obstructive Pulmonary Disease	Diabetes	≥2 Chronic Diseases
**7-day**	25.9 (23.9–28.1)	34.2 (32.4–35.9)	29.5 (25.4– 33.7)	23.5 (16.6–31.7)	24.3 (19.2–30.0)	25.1 (20.4–30.3)	24.7 (21.2–28.5)
**30-day**	22.3 (20.4–24.4)	29.7 (28.0–31.3)	25.6 (21.8–29.7)	20.5 (13.9–28.4)	19.7 (15.0–25.1)	21.6 (17.2–26.6)	21.6 (18.3–25.3)

In multivariate analyses of 30-day quit rates among respondents to the 7-month follow-up survey, callers who had any chronic disease were less likely to quit than callers who had no chronic disease. African American callers, callers who had Medicaid insurance, and callers who smoked more cigarettes per day were each less likely to quit. Callers who had more than a high school education and callers who completed more calls were more likely to quit. We found similar results among 7-month survey respondents who were enrolled in the multicall program (Table 2). In a comparison of callers who had no disease and callers who had only 1 disease (eg, asthma only) or 2 or more diseases, having a chronic disease was associated with lower quit rates (Wald χ^2^_5_ = 20.3, *P* = .001), even after controlling for covariates and state (Table 3). We found similar results in analyses of 7-day quit rates.

**Table 2:** Comparison of “Any Chronic Disease” and “No Disease Groups” for 30-Day Quit Rates Among Callers Who Participated in 7-Month Follow-Up Survey and Multicall Counseling Program (n = 3,250)^a^

Effect	Odds Ratio (95% CI)	*P* Value
Any chronic disease	0.81 (0.68–0.96)	.02
No chronic disease	1 [Reference]	
**Race^b^**		
African American	0.73 (0.58–0.91)	.04
Other	0.89 (0.67–1.91)	.74
White	1 [Reference]	
**Insurance status**		
Medicaid	0.69 (0.54–0.88)	.003
Uninsured	0.93 (0.76–1.15)	.23
Other insurance	1 [Reference]	
**Education**		
>High school	1.22 (1.04–1.44)	.02
≥High school	1 [Reference]	
**Nicotine replacement therapy (patch or gum)**		
Used	0.93 (0.70–1.24)	.61
Did not use	1 [Reference]	
**Other^c^**		
No. of cigarettes per day	0.98 (0.98–0.99)	<.001
No. of calls completed	1.05 (1.02–1.09)	.003

Abbreviation: CI, confidence interval.

^a^ The survey asked about the following 4 chronic diseases only: asthma, CAD, COPD, and diabetes. Adjusted logistic regression model included all variables in the table, plus age, sex, and state.

^b^ Each race category includes Hispanics and non-Hispanics.

^c^ Odds ratios represent coefficients for these continuous variables.

**Table 3:** Comparison of 6 Disease Groups for 30-Day Quit Rates Among Callers Who Participated in 7-Month Follow-Up Survey and Multicall Counseling Program (n = 3,250)^a^

Effect	Odds Ratio (95% CI)	*P* Value
**Disease group**		
Asthma only	0.84 (0.65–1.10)	.91
CAD only	0.86 (0.50–1.48)	.88
COPD only	0.71 (0.48–1.06)	.37
Diabetes only	0.79 (0.55–1.13)	.75
≥2 diseases	0.81 (0.61–1.07)	.81
No chronic disease ^b^	1 [Reference]	
**Race^b^**		
African American	0.72 (0.57–0 .91)	.03
Other	0.90 (0.67–1.20)	.71
White	1 [Reference]	
**Insurance status**		
Medicaid	0.69 (0.54–0.88)	.003
Uninsured	.93 (0.76–1.15)	.23
Other insurance	1 [Reference]	
**Education**		
>High school	1.22 (1.04–1.44)	.02
≥High school	1 [Reference]	
**Nicotine replacement therapy (patch or gum)**		
Used	0.93 (0.70–1.24)	.61
Did not use	1 [Reference]	
**Other**		
No. of cigarettes per day ^c^	0.98 (0.98–0.99)	<.001
No. of calls completed^c^	1.06 (1.02–1.09)	.004

Abbreviation: CI, confidence interval; CAD, coronary artery disease; COPD, chronic obstructive pulmonary disease.

^a^ Having a chronic disease was associated with lower quit rates, even after controlling for covariates and state (Wald χ^2^_5_ = 20.3, *P* = .001). The survey asked about the following 4 chronic diseases only: asthma, CAD, COPD, and diabetes. Logistic regression model included all variables above plus age and sex, which were not significant, and state.

^b^ Each race category includes Hispanics and non-Hispanics.

^c^ Odds ratios represent coefficients for these continuous variables.

## Discussion

This study contributes to the limited data available on the use of state tobacco quitlines and quit rates among people who have chronic diseases. In our study, one-third of tobacco users enrolled in 15 state quitlines had at least 1 of 4 selected chronic diseases. Although we found that callers who had a chronic disease were less likely to quit tobacco than callers who had none of the 4 diseases, another study reported higher quit rates among people who had smoking-related diseases (18). Smokers in that study, however, were not receiving cessation treatment. The quit rates found in our study (19.7%–29.7%) are within the range (16%–36%) observed in other state quitlines (9,19). The 30-day quit rates among callers who had diabetes (21.6%) and COPD (19.7%) in our study are higher than rates found in studies of primary-care–based smoking cessation interventions: 17% among smokers who had diabetes (20) and 16% among smokers who had COPD (21). Demographic differences by chronic disease status (eg, older age, more years of smoking) could explain some of the variability in quit rates.

Although quitline callers who had any chronic disease were more likely to use tobacco within 5 minutes of waking (ie, they had a greater level of nicotine addiction) than callers who had no disease, they were less likely to receive NRT, even though none of the selected diseases contraindicates NRT. The Public Health Service Clinical Practice Guideline states that NRT should be used with caution by those in the immediate postmyocardial infarction period, those who have serious arrhythmias, and those who have unstable angina pectoris (9). The Centers for Disease Control and Prevention (CDC) recommends that quitlines be funded to provide 8 weeks of NRT to people who have Medicaid or no health insurance and 2 weeks to others (22). Research is needed to determine whether tobacco users who have a chronic disease are less likely than tobacco users with none of these diseases to receive NRT through quitlines because of state eligibility requirements, caller refusal, or other factors, such as medical advice.

Although two-thirds of callers in this study had enrolled in the multicall counseling program, they completed on average only 2 or 3 counseling calls, which is consistent with the general quitline population (23). In addition, although we found that quit rates increased as the number of completed calls increased, we found that callers who had a chronic disease completed more calls than callers who had no disease, but they had lower quit rates. These findings are inconsistent with research showing that abstinence increases with treatment intensity (9,24,25). In our study, however, statistical significance could have resulted from the large sample size; the number of calls completed differed by less than 1 call (0.3 to 0.4 calls) among groups. Further research is needed to examine this apparent discrepancy.

In summary, we observed differences in characteristics of quitline callers according to chronic disease. Callers who had asthma only were younger, callers who had COPD only were more likely to be non-Hispanic white, and callers who had COPD only or 2 or more chronic diseases were more likely to use tobacco within 5 minutes of waking. These and other differences suggest future efforts are needed to improve use and effectiveness of treatment in these populations.

This study had several limitations. First, we studied only 4 chronic diseases; we selected these 4 because they are routinely asked about during quitline enrollment. Tobacco users who have any of these 4 diseases are considered to have greater health risks than nonsmokers (6).

Second, we relied on self-reported disease status, which may have resulted in under- or overreporting (eg, some people may mistake their smoking-induced respiratory symptoms for symptoms of asthma). However, studies show that self-report adequately represents disease status for epidemiological purposes (26). Self-reported smoking status could also have been subject to bias, but studies show that self-reported abstinence adequately describes population-level smoking rates (27). Third, we pooled follow-up data among different sampling frames from 9 states. We were not able to calculate follow-up survey response rates or intent-to-treat quit rates because we did not have access to the denominators (ie, the number sampled for the segment of follow-up that overlapped with our study timeline). In general, survey response rates reported by these quitlines range from 34% to 52%. Fourth, quitlines offered different levels of services during the study, and this variable might have influenced enrollment and outcomes. Offering free NRT via state quitlines can increase the number of calls to the quitline, reach larger proportions of certain populations (28,29), and improve outcomes (30,31). By adding state to the multivariate analyses, we attempted to control for the difference among states in quitline services. Finally, using different criteria for selecting variables to include in statistical models (eg, *P* values > .05) may have produced additional covariates for modeling.

Despite these limitations, this study offers new information about the relationship between chronic disease and quitline services and points out the need to integrate cessation treatment into the medical management of chronic diseases (22,32–34). Smokers who have a smoking-related chronic disease and quit have a lower risk for death from the disease than those who continue to smoke (7). Quitlines are an effective population-based cessation intervention that are available for free in all states and can be easily integrated into chronic disease programs by encouraging referrals to quitlines when tobacco users are identified in screening and preventive care programs (22). In a recent survey of quitlines, all but 2 of the 21 states responding noted their quitline had worked with chronic disease programs to add tobacco treatment (19). For example, in 2010, the CDC’s breast, cervical, and colorectal cancer screening programs began screening for tobacco use; for people who expressed an interest in quitting, the programs made referrals to community-based cessation programs, including quitlines. Coordinated tobacco control efforts have been shown to increase awareness of quitlines by chronic disease staff and increase the number of fax referrals to quitlines (35). The PHS Clinical Practice Guideline recommends that clinicians identify smokers, provide brief counseling, and make referrals to cessation services and that health care systems facilitate patient access to quitlines and promote their use (9). Resources are needed for state quitlines to ensure that comprehensive, proactive counseling and NRT are available to all tobacco users who seek assistance with quitting.

The findings of this study also indicate quitlines may need to offer more intensive treatment for callers who have with a chronic disease than for callers who do not have a chronic disease. Such treatment may include re-enrollment, longer duration of NRT, and combination therapy (nicotine patch and gum). Research indicates that intensive or combined pharmacological treatments are more cost-effective when delivered to high-risk populations (36).
